# ENO1 as a Central Regulator Linking Metabolic Reprogramming to Tumor Plasticity

**DOI:** 10.3390/ijms27104479

**Published:** 2026-05-16

**Authors:** Tsung-Chieh Lin

**Affiliations:** 1 Genomic Medicine Core Laboratory, Department of Medical Research and Development, Chang Gung Memorial Hospital, Linkou, Taoyuan City 333, Taiwan; tclin1980@cgmh.org.tw; Tel.: +886-3-3281200 (ext. 7722); 2 Department of Biomedical Sciences, Chang Gung University, Taoyuan City 333, Taiwan

**Keywords:** ENO1, cancer metabolism, tumor progression, metastasis, drug resistance, prognostic biomarker, pan-cancer analysis

## Abstract

Alpha-enolase (ENO1) is a multifunctional protein best known for its canonical role in glycolysis, but growing evidence indicates that it also plays important roles in cancer development and progression. This review summarizes the current knowledge regarding the biological and clinical significance of ENO1 across multiple cancer types. We first outline the physiological characteristics of ENO1 and its distribution in normal tissues. Then, we discuss its aberrant expression patterns and genomic alterations in human cancers. We further examine the evidence linking ENO1 to major cancer-related processes, including proliferation, apoptosis resistance, cancer stemness, autophagy, metastasis, drug resistance, and angiogenesis. In addition, we review studies that evaluate the association between ENO1 expression and patient prognosis in pan-cancer datasets and individual malignancies. Collectively, the available literature indicates that ENO1 is closely associated with malignant progression through its involvement in metabolic reprogramming and broader tumor-promoting cellular functions. These findings suggest that ENO1 may serve as a context-dependent biomarker and a candidate therapeutic target in selected cancer settings; however, further mechanistic validation and clinically annotated studies are required before its translational value can be firmly established.

## 1. Introduction

**Figure 1:**
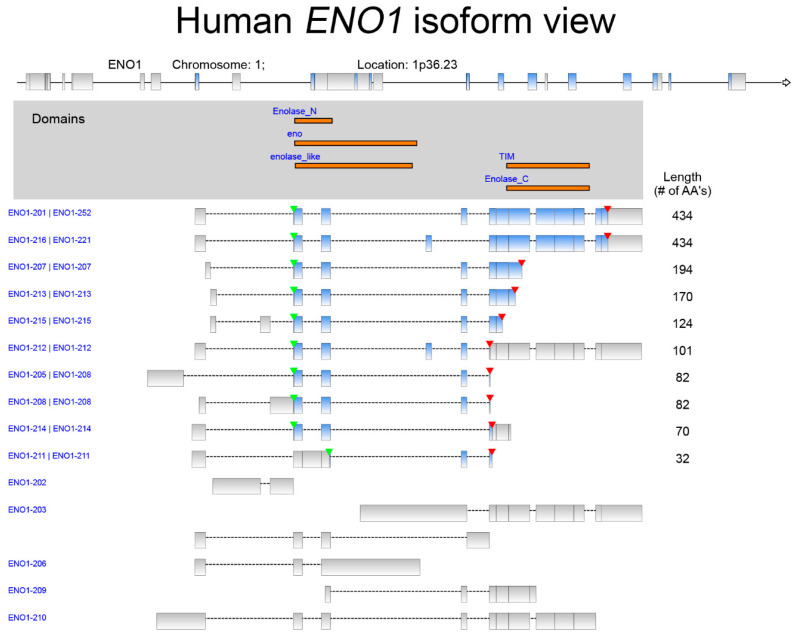
The isoform view of human *ENO1*. The red and green arrowheads indicate the positions of the stop codon and the transcription start site, respectively. The matched protein domains among those isoforms are displayed in orange. The information is based on data adapted with permission from the Ingenuity Pathway Analysis (accessed on: 2 February 2026). Copyright Year 2026, QIAGEN.

ENO1 (alpha-enolase) is encoded by the *ENO1* gene and catalyzes the conversion of 2-phosphoglycerate to phosphoenolpyruvate in glycolysis, making it a central determinant of carbon flux toward ATP and biosynthetic intermediates [1]. The *ENO1* gene is located at 1p36.23 in humans and encodes various isoforms, with a total of 15 transcript variants (Figure 1). The alternative translation of the *ENO1* gene may lead to the synthesis of a shorter protein variant called the c-MYC promoter-binding protein (MBP-1) that acts as a transcription repressor for the *c-MYC* oncogene [2]. ENO1 is best known for its role in modulating glycolysis and embryonic stem cell differentiation [3].

Recent studies have highlighted a broader role for ENO1 in tumorigenesis and cancer progression. In a study of chronic hepatitis B virus (HBV) infection-associated hepatocellular carcinoma cells, ENO1 overexpression causes malignant behaviors in cancer, and this increased expression is induced by the hepatitis B virus X protein (Hbx) [4]. In a study addressing the role of long non-coding RNA LINC00183 in colorectal cancer proliferation and invasiveness, the LINC00183–enolase 1 interaction was uncovered, leading to the activation of glycolysis, lactate accumulation, and upregulation of the oncogene GDF15 [5]. Enolase 1 could directly bind and stabilize BACE2 from lysosomal-dependent degradation to remodel cholesterol metabolism, thereby promoting liver cancer progression [6]. Growing experimental evidence indicates that ENO1 and its downstream signaling network contribute to multiple malignant phenotypes, including angiogenesis, metastasis, proliferation, cancer stemness, and drug resistance, thereby promoting tumor development. Furthermore, enolase 1 has been targeted for cancer cell-specific drug delivery in a colon cancer study. The enhanced therapeutic efficacy and selectivity of gemcitabine conjugated with the ENO1-targeting peptide (GCB-P) was reported [7]. Therefore, the objective of this review is to provide an evidence-based synthesis of ENO1 in cancer biology by integrating data on normal tissue distribution, somatic alterations, tumor expression patterns, cancer-associated cellular functions, and clinical outcome correlations. Particular attention is given to distinguishing well-supported roles, such as glycolytic reprogramming, proliferation, metastasis, and treatment resistance, from areas where current evidence remains limited or indirect, including angiogenesis and lineage plasticity.

## 2. ENO1 Somatic Mutation and Cancer

**Figure 2:**
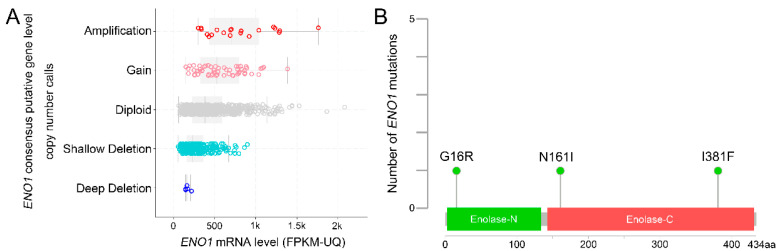
(**A**) The results of a pan-cancer study enrolling 991 samples using whole genome data reveals the types, copy number variations, and sites of *ENO1* mutations. (**B**) Light green indicates the missense mutations. Data were adapted with permission from cBioPortal (https://docs.cbioportal.org/user-guide/faq/#can-i-use-figures-from-the-cbioportal-in-my-publications-or-presentations) and accessed on 4 February 2026.

Classical recurrent activating point mutations of *ENO1* are not commonly reported as canonical oncogenic drivers. The results of a pan-cancer study that analyzed 991 samples revealed data in both the profiles of copy number variation, including the presence of one or more extra copies, and mutational status (Figure 2 and Table 1) [8,9]. In addition, *ENO1* alterations of translational relevance often reflect copy number loss or deletion that creates therapeutic dependencies. In *ENO1*-deleted cancers, small-molecule strategies that target enolase biology have been explored as a way to exploit collateral lethality and metabolic liabilities, with glioblastoma-related models serving as a proof-of-concept for chemical targeting approaches [10]. The direct experimental validation of the functional effects of G16R, N161I, and I381F is currently lacking. Hence, current pan-cancer mutation data might indicate that it remains to be determined whether ENO1 is commonly affected by recurrent hotspot mutations and functions as a classical mutation-driven oncogene.

## 3. ENO1 Distribution and Expression in Normal Cell Types

**Figure 3:**
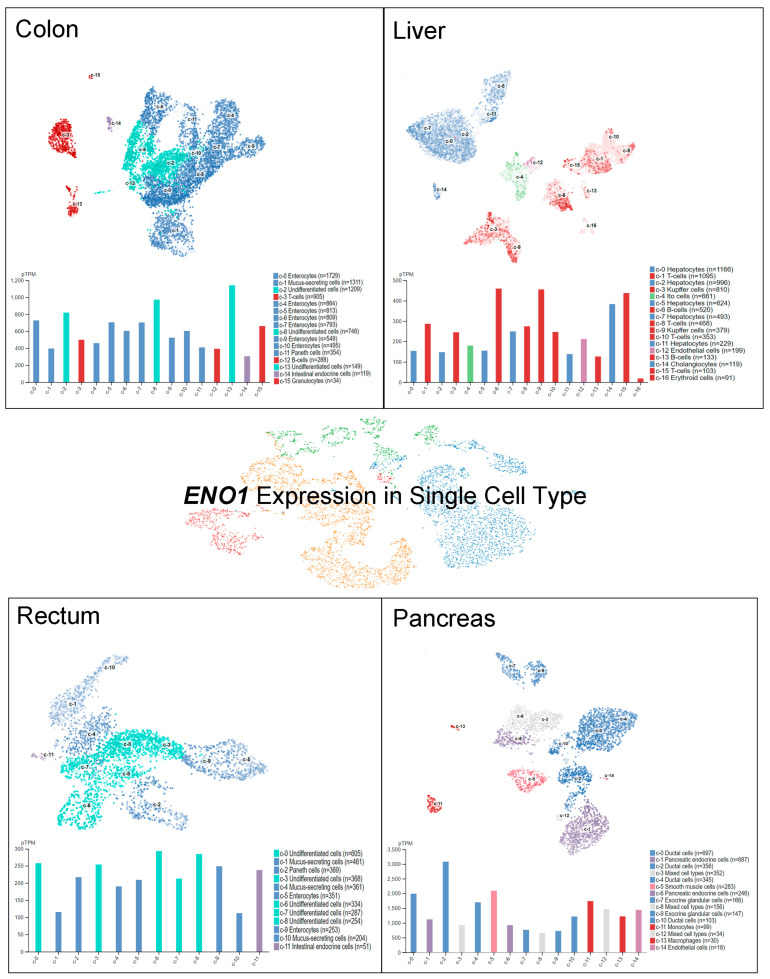
Human *ENO1* expression at the single-cell level in 192 specific cell types. The *ENO1* level was detected by scRNA-seq in various tissues. The RNA expression levels in the cell type clusters were identified in the indicated tissue and visualized by the Uniform Manifold Approximation and Projection (UMAP) plot. Data were adapted with permission from the Human Protein Atlas (HPA) database (https://www.proteinatlas.org/about/licence#citation_guidelines_for_the_human_protein_atlas, accessed on: 2 February 2026).

*ENO1* has been investigated by single-cell RNA sequencing (scRNA-seq) to explore its RNA distribution among specific cell types in a given tissue [11,12,13,14]. Importantly, single-cell RNA expression might shed light on the results found in studies focusing on cancer biomarker identification and sites of tumorigenesis [15]. A cell type atlas provides the scRNA-seq data of the specific gene expression in 192 specialized clusters/cell types (Human Protein Atlas, https://www.proteinatlas.org) [16]. ENO1 expression levels in normal organs, namely, the colon, liver, rectum, and pancreas, are indicated at the single-cell level in Figure 3. A relatively higher ENO1 expression has been detected in undifferentiated cells, enterocytes, and granulocytes in the colon. B cells, Kupffer cells, T cells, and cholangiocytes in the liver have been found to display significant *ENO1* expression. In addition, *ENO1* expression in the rectum has been specifically observed in undifferentiated cells, enterocytes, and intestinal endocrine cells, but not in other cell types. In pancreatic tissues, *ENO1* RNA expression has been found in ductal cells, smooth muscle cells, and monocytes. These research findings suggest the potential sites of ENO1-mediated downstream events that may be relevant to tumorigenesis.

## 4. ENO1 Expression in Cancers

**Figure 4:**
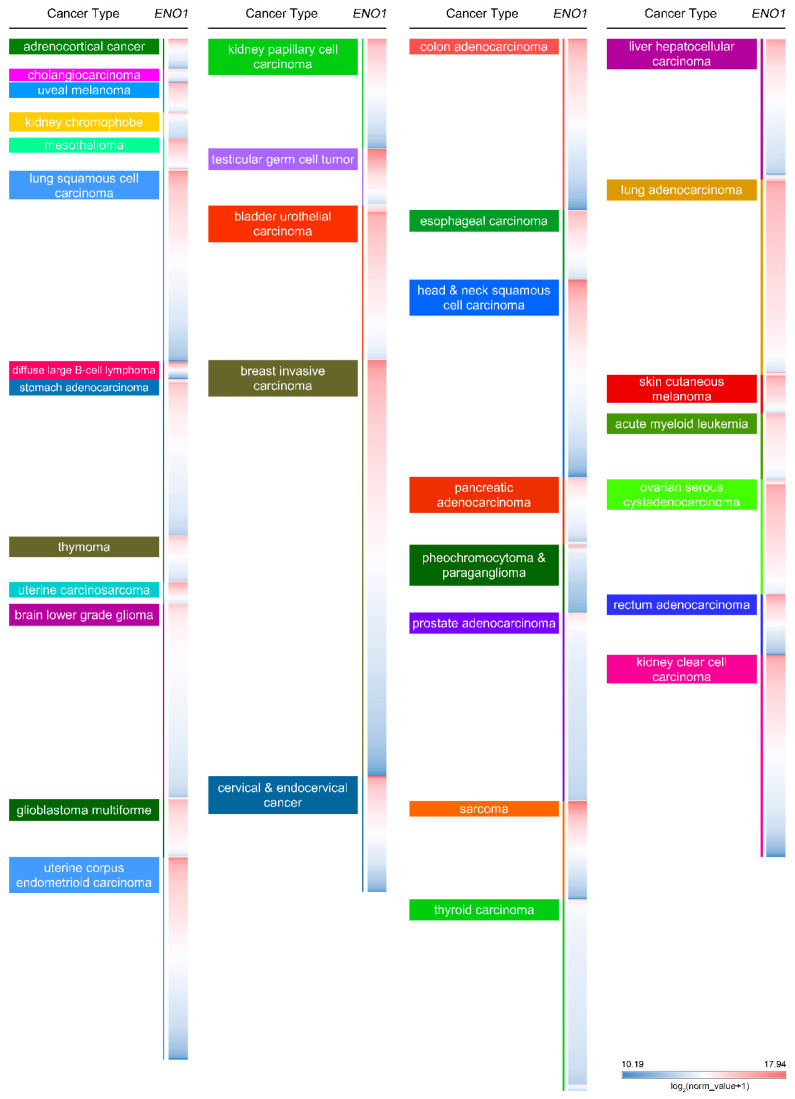
A view of *ENO1* expression levels in pan-cancer. In a pan-cancer dataset, *ENO1* expression levels are displayed separately for 32 types of cancer. The red and blue colors in the heat map highlight the high and low expression of *ENO1*, respectively. The data were retrieved from The Cancer Genome Atlas (TCGA) database and analyzed (accessed on: 2 February 2026).

In tumor tissues, ENO1 expression is frequently elevated alongside glycolytic reprogramming. Immunohistochemical ENO1 staining has been proposed as a useful diagnostic or pathologic adjunct in resected lung adenocarcinoma specimens [1]. In a study of intrahepatic cholangiocarcinoma, ENO1 is found to be highly expressed in tumor tissues, and its level is stabilized by inhibiting K63-linked ubiquitination and lysosome-mediated degradation while interacting with PSMD14 [17]. In triple-negative breast cancer cells, lactylation of enolase 1 has been shown to inhibit lysosome-mediated degradation [18]. In addition, enolase 1 degradation could be induced after direct binding with a small molecule inhibitor, SU212, in triple-negative breast cancer [19]. A colorectal cancer study performing RNA immunoprecipitation and RNA pull down assays shows the interaction between enolase 1 and long non-coding RNA LINC00183. This interaction protects enolase 1 from ubiquitin–proteasome-mediated degradation because of the mask that occurs at a critical K262 residue [5]. In hepatocellular carcinoma, the proteasomal degradation via a ubiquitin-dependent axis has also been reported to be facilitated by direct binding with KBTBD11 [20]. Moreover, enolase 1 stability is found to be controlled by the direct binding with carcinoembryonic antigen-related cell adhesion molecule 6 (CEACAM6) in bladder cancer [21]. In addition, enolase 1 could be stabilized by desumoylation via direct interaction with SENP1 [22]. In a cervical cancer study, the upregulation of ENO1 and the SIX1 binding site at the ENO1 promoter region have been uncovered [23]. In addition, the increase in circulating ENO-1^+^CD81^+^ extracellular vesicles could be detected from the serum of Ewing sarcoma patients [24]. *ENO1* levels in a pan-cancer panel were shown in integrated clinical information and transcriptomic studies (University of California, Santa Cruz, n = 12,839) [25]. As seen in Figure 4, *ENO1* was shown to be highly upregulated in uveal melanoma, mesothelioma, uterine carcinosarcoma, glioblastoma multiforme, testicular germ cell tumor, bladder urothelial carcinoma, lung adenocarcinoma, skin cutaneous melanoma, acute myeloid leukemia, and ovarian serous cystadenocarcinoma. In contrast, lower *ENO1* levels were detected in kidney chromophobe, brain lower grade glioma, pheochromocytoma and paraganglioma, prostate adenocarcinoma, and thyroid carcinoma.

## 5. ENO1 Correlation with Clinical Outcome

Clinical associations between ENO1 and various outcomes are frequently reported, but directionality can be influenced by stage, treatment context, and ENO1 compartmentalization. In diffuse large B-cell lymphoma, a hub gene signature including ENO1 has been identified as predicting worse clinical outcomes [26]. ENO1 was discovered to be closely associated with poor intrahepatic cholangiocarcinoma prognosis [17]. In lung adenocarcinoma, ENO1 immunohistochemical expression has been examined for pathologic diagnosis and potential prognostic utility in resected tumors [1]. In breast cancer, ENO1 sub-localization patterns have been analyzed in benign versus malignant lesions, suggesting that where ENO1 resides may matter when interpreting tumor aggressiveness [27]. Furthermore, mechanistic work links ENO1-related pathways to radiosensitivity in breast cancer through ENO1 ubiquitination control [19]. In hepatocellular carcinoma, therapeutic strategies that promote ENO1 degradation have been reported to augment anti-programmed cell death protein 1 (PD-1) responses, supporting the notion that ENO1 is a targetable determinant of outcomes in immunotherapy settings [20]. In a pancreatic cancer study, high ENO1 levels have been reported to correlate with a poorer prognosis in patients [28]. In bladder cancer, ENO1-linked regulation of programmed death-ligand 1 (PD-L1) and immunotherapy response have been reported, illustrating how ENO1-centered programs may translate into clinically actionable immune phenotypes [29]. Finally, in radiotherapy contexts, targeting ENO1 to reprogram macrophage polarization has been reported to enhance the effect of radiotherapy, therefore connecting ENO1 biology to treatment outcome via microenvironmental remodeling [30]. Comprehensive pan-cancer studies integrating cancer patients’ clinical data with *ENO1* RNA expression profiles, namely the Human Protein Atlas (HPA) [16,31,32,33,34] and the Kaplan–Meier plotter databases [35], have been completed. The prognostic data of *ENO1* in different cancer types are listed in Table 2. Data were adapted with permission from the HPA database https://www.proteinatlas.org/about/licence#citation_guidelines_for_the_human_protein_atlas (accessed on 1 February 2026). *ENO1* appears to be an inferior prognostic biomarker in cohorts of patients with glioma, lung cancer, head and neck cancer, liver cancer, pancreatic cancer, urothelial cancer, breast cancer, and cervical cancer. Furthermore, in patients diagnosed with breast and gastric cancer, high *ENO1* expression levels determined by array are correlated with poor clinical outcomes. The prognostic value of ENO1 should be interpreted as context dependent rather than universal, because its clinical performance may be affected by tumor type, assay platform, cutoff selection, tumor purity, subcellular localization, immune infiltration, and survival endpoint.

## 6. ENO1 and Proliferation

Evidence supporting pro-proliferative and anti-apoptotic roles of ENO1 has been reported in several epithelial malignancies. ENO1 overexpression in hepatocellular carcinoma leads to the increase in cancer cell proliferation as well as the inhibition of apoptosis [4]. Furthermore, ENO1 has been found to be a direct binding target of rupestonic acid, which exerts its anti-tumor proliferation effect on hepatocellular carcinoma [36]. Importantly, enolase-associated glycolysis could be suppressed by the direct interaction with KBTBD11 that leads to hepatocellular carcinoma apoptosis [20]. In addition, activation of the HDAC7/ENO1 signaling axis was found in hepatocellular carcinoma proliferation due to a deficiency in ARID1A [37]. In intrahepatic cholangiocarcinoma, ENO1 is a mediator of the L-lactate/PSMD14 axis that modulates ferroptosis resistance [17]. In gastric cancer systems, ENO1 activity has been linked to an accelerated glycolytic pathway and increased survival under treatment pressure, with ENO1-driven metabolic output implicated in chemoresistance and growth advantages [38,39]. Consistent with a growth-supporting role, shRNA-mediated suppression of ENO1 in gastric cancer models reduced proliferation and promoted apoptosis [40], while transcriptomic profiling after ENO1 knockdown highlighted broad gene-expression shifts compatible with reduced malignant potential [41]. In lung cancer models, ENO1 can enhance proliferative and invasive features through upregulation of PGC1α and CXCR4 [42]. In a cervical cancer study, the transcriptional activation of ENO1 could be triggered by SIX1, thereby enhancing cancer cell proliferation and aerobic glycolysis [23]. Silencing ENO1 expression in multiple myeloma could repress tumorigenicity and cause cell cycle arrest. Meanwhile, sensitization of tumor cells to apoptosis occurs via modulation of mitophagy [43]. Context-dependent observations can arise when ENO1 is perturbed indirectly through upstream regulators or when cell death programs dominate. Recent breast cancer studies connect altered glycolysis control and stress signaling to tumor phenotypes in which ENO1 sits within broader hypoxia–hypoxia-inducible factor 1-alpha (HIF-1α) circuitry [44]. Results of another breast cancer study show that silencing ENO1 could result in autophagy-related ferroptosis via modulating CST1 [45]. Additionally, in peripheral T-cell lymphoma models, ENO1 knockdown-linked pathways have been connected to ferroptosis and anti-tumor immunity under epigenetic therapy, illustrating how ENO1-associated metabolic wiring can intersect with cell death modalities rather than uniformly suppress them [46].

## 7. ENO1 and Cancer Stemness

ENO1 has been linked to stem-like phenotypes, especially where glycolysis supports self-renewal and stress tolerance. In lung cancer stem cell models, ENO1 promoted self-renewal and malignant behavior through AMP-activated protein kinase (AMPK)/mechanistic target of rapamycin (mTOR) signaling, providing a mechanistic bridge between metabolic state and stemness-associated programs [47]. Quiescent self-renewal of leukemia stem cells has been considered the main factor leading to progression in acute myeloid leukemia. Trajectory analysis of single-cell RNA sequencing data reveals high ENO1-expressing cancer cells in the initial stages of differentiation. Meanwhile, upregulation of ENO1 and stemness-related genes could be identified in malignant cells during early differentiation. Furthermore, cancer cell differentiation occurs after knockdown of ENO1 [48]. Stemness-associated metabolic rewiring can also be reinforced by therapy selection. For example, colorectal cancer drug resistance accompanied by epithelial–mesenchymal transition (EMT) programs, which is considered a frequent correlate of stemness, has been associated with ENO1-dependent states [49]. In a study of gastric cancer, enolase 1 could promote adenosine triphosphate (ATP) and lactate production by modulating glycolysis as well as the AMPK/mTOR and phosphoinositide 3-kinase (PI3K)/AKT axis to drive EMT-related marker expression, cancer stemness, and self-renewal [50]. Although direct evidence for ENO1-driven trans-differentiation is limited in the available primary set, ENO1’s ability to couple metabolic flux to lineage programs suggests that lineage plasticity could be indirectly favored when ENO1 sustains glycolytic and redox demands during selection pressures. Although several studies support a role for ENO1 in maintaining stem-like phenotypes, whether ENO1 acts as a direct cancer stemness driver or as a metabolic consequence of stem-like adaptation remains unresolved. In lung cancer stem cell models, ENO1 promoted self-renewal and malignant behavior through AMPK/mTOR signaling, whereas in acute myeloid leukemia, single-cell trajectory analysis suggested that ENO1-high malignant cells were enriched at early differentiation states and that ENO1 knockdown promoted differentiation. These findings support a functional link between ENO1 and stemness. However, in colorectal and gastric cancer, the association between ENO1 and stemness is often intertwined with EMT, drug resistance, glycolytic rewiring, and therapy selection pressure. Thus, ENO1 may not uniformly initiate stemness across all tumor types; rather, it may sustain the metabolic and stress-tolerant state required for stem-like cells to persist under adverse microenvironmental or therapeutic conditions. Future studies should distinguish true cancer stemness from EMT-associated plasticity by using standardized sphere-formation assays and limiting dilution transplantation, as well as by performing lineage tracing, single-cell multi-omics, and rescue experiments after ENO1 perturbation.

## 8. ENO1 and Autophagy

Autophagy can either buffer metabolic stress or contribute to therapy adaptation, and recent work connects ENO1-centered metabolism to autophagy-related phenotypes. In breast cancer, regulators that converge on ENO1 have been reported to coordinate autophagy and ferroptosis, positioning ENO1 within stress-response networks that can shape tumor cell survival [45]. In glioblastoma models, autophagy-dependent secretion of ENO1 has been proposed to remodel the tumor microenvironment toward immunosuppression, suggesting that autophagy can influence not only intracellular ENO1 functions but also extracellular ENO1 availability [51]. In chronic myeloid leukemia, an ENO1 blockade increases tyrosine kinase inhibitor (TKI) sensitivity and promotes ferroptosis susceptibility in TKI-resistant cells by AMPK/mTOR pathway-mediated GPX4 autophagic degradation [52].

## 9. ENO1 and Metastasis

Metastatic progression requires coordinated energy production, cytoskeletal remodeling, and microenvironmental interactions, all of which can intersect with ENO1 biology. In hepatocellular carcinoma, exosome-derived ENO1 has been shown to promote growth and metastasis, supporting a model in which vesicle-associated ENO1 helps propagate pro-metastatic signaling between tumor cells and the microenvironment [53]. The enhancement in cancer cell migration, invasion, and HBV replication was reported to be induced by ENO1 overexpression [4]. In breast cancer, ENO1 has been linked to increased invasiveness [54]. A bioinformatic approach using available databases reveals that ENO1 is a biomarker for predicting breast cancer metastasis and progression [54]. Moreover, enolase 1 is a direct interacting target of SU212, a small molecule inhibitor, and exerts its downstream anti-tumor effects, including glucose uptake, glycolytic activity, and metastasis, in a patient-derived xenograft model of triple-negative breast cancer [19]. The increase in breast cancer metastasis by way of the EMC2/USP7/ENO1 axis has been addressed. Enolase 1 could be stabilized by scaffold protein EMC2-mediated deubiquitylation; it also activates the downstream MYB/PDK1/AKT (T308)/mTOR (S2448) signaling pathway [55]. In colorectal cancer, ENO1-associated pathways can promote metastasis [56]. Further, post-translational modification such as the O-GlcNAcylation of ENO1 has been reported to drive progression [57], complementing mechanistic links between ENO1 and AMPK/mTOR-associated metastatic phenotypes [58]. In addition, the PPARγ/ENO1 signaling axis and glycolysis are involved in conducting pachymic acid-mediated suppression in colon cancer invasion [59]. In bladder cancer, ENO1 is pivotal in promoting cancer cell migration and invasion via direct interaction with CEACAM6, which is a well-known molecule that controls cancer metastasis [21]. In a study of macrophage polarization in lung cancer, ENO1 could form an RNA-protein complex with CircFUT8 and facilitate M2 macrophage polarization and cancer progression, including cell migration and invasion [60]. Furthermore, in an osteosarcoma study conducted by single-cell RNA-sequencing, ENO1 was found to be overexpressed at the lung metastasis site. Knockdown of ENO1 impedes cancer cell migration/invasion and metastasis in cell and animal models, respectively [61]. In a metastatic study investigating intrahepatic cholangiocarcinoma, ENO1 could be stabilized by DCDC2, which induces AKT phosphorylation and immune evasion by impairing CD8^+^ T cell functionality [62]. Furthermore, pancreatic cancer migration, invasion, and glycolytic flux are reported to be enhanced by forced co-expression of ENO1 and PGM2L1 [63]. We established an integrative model consisting of activating and inhibitory regulatory arms. The activating arm includes ENO1 overexpression, stabilization, post-translational modification, exosomal transfer, surface-associated plasminogen receptor activity, immune evasion, macrophage polarization, and extracellular matrix remodeling. The inhibitory arm includes ENO1 degradation, pharmacological inhibition, antibody-mediated blockade, and suppression of ENO1-related glycolysis.

## 10. ENO1 and Drug Resistance

Several primary studies link ENO1 to chemotherapy resistance through metabolic buffering, EMT, and nucleotide or redox homeostasis. In gastric cancer, ENO1-driven glycolysis has been reported to support chemoresistance [38], and ENO1 ubiquitination status can modulate therapeutic sensitivity, as illustrated by work connecting ENO1 ubiquitination to the palbociclib response in gastric cancer models [64]. Given the shared features of glycolysis and lactate accumulation in cancers, ENO1 lactylation is further characterized as a feedback loop for augmenting osimertinib resistance in lung adenocarcinoma [65]. In gastric cancer, cisplatin resistance as well as cancer progression could be driven by the desumoylation of enolase 1 by SENP1 [22]. In colorectal cancer, ENO1 has been implicated in 5-fluorouracil resistance in association with EMT programs [49]. In pancreatic cancer, ENO1-mediated metabolic and nucleotide programs have been connected to gemcitabine resistance via the stabilization of RRM2 and altered deoxycytidine synthesis [66]. In pancreatic cancer, knockdown of ENO1 results in increased sensitivity to gemcitabine through MYC/RRM1-mediated glycolysis [28]. Enolase 1 has been reported to elicit bortezomib resistance in multiple myeloma through the YWHAZ/Parkin signaling pathway to activate mitophagy [43]. In bone metastatic prostate cancer, the treatment of ENO1-targeting inhibitor phosphonoacetohydroxamate (PhAH) leads to the restoration of cancer cell sensitivity to enzalutamide (ENZ) [67]. Context-dependent inversions can also occur when ENO1 is genetically absent. In ENO1-deleted tumor settings, as reported in glioblastoma-related collateral lethality frameworks, chemical targeting strategies can exploit dependencies that emerge from ENO1 loss rather than ENO1 abundance, making ENO1 status a predictive biomarker for targeted vulnerability [10]. ENO1 expression or post-translational regulation may serve as a predictive marker of resistance to chemotherapy. From a clinical trial perspective, ENO1-targeted therapy remains in its early stages, but it is increasingly translational. HuL001/HL217 is a humanized ENO1-blocking antibody developed to target cell-surface ENO1 and inhibit plasmin activation, migration, invasion, glycolysis, lactate secretion, and tumor microenvironment remodeling. A phase I study of HuL001 was registered to evaluate the safety, tolerability, pharmacokinetics, and immunogenicity in healthy volunteers and idiopathic pulmonary fibrosis subjects. Nevertheless, the clinical relevance of ENO1 targeting remains to be established. Most evidence still comes from preclinical models, and current clinical studies have not yet demonstrated definitive efficacy endpoints in randomized cancer trials.

## 11. ENO1 and Angiogenesis

Direct ENO1-specific angiogenesis mechanisms are less frequently demonstrated than the effects on proliferation or invasion, but ENO1-centered glycolysis can plausibly support angiogenesis indirectly by sustaining hypoxia responses and lactate-rich microenvironments. In breast cancer, stress and hypoxia signaling that intersects with glycolysis can influence ENO1-associated metabolic states [44]. In metastatic breast tumors, glycolysis-related genes including ENO1 are linked to hypoxia and angiogenesis [68]. In a prostate cancer study, ENO1 mAb was found to inhibit vascular endothelial growth factor A (VEGF-A)-induced tube formation of endothelial cells in vitro, and to target multiple tumor microenvironment (TME) niches involved in prostate cancer bone metastasis via a plasmin-related mechanism [69]. Overall, the current evidence supports an angiogenesis-related or microenvironment-modifying role of ENO1, but it does not offer a definitive conclusion that ENO1 is an independent angiogenic ligand across cancer types. For this reason, ENO1-associated angiogenesis should be integrated with tumor microenvironment remodeling unless stronger causal evidence becomes available.

## 12. Summary and Perspectives

**Figure 5:**
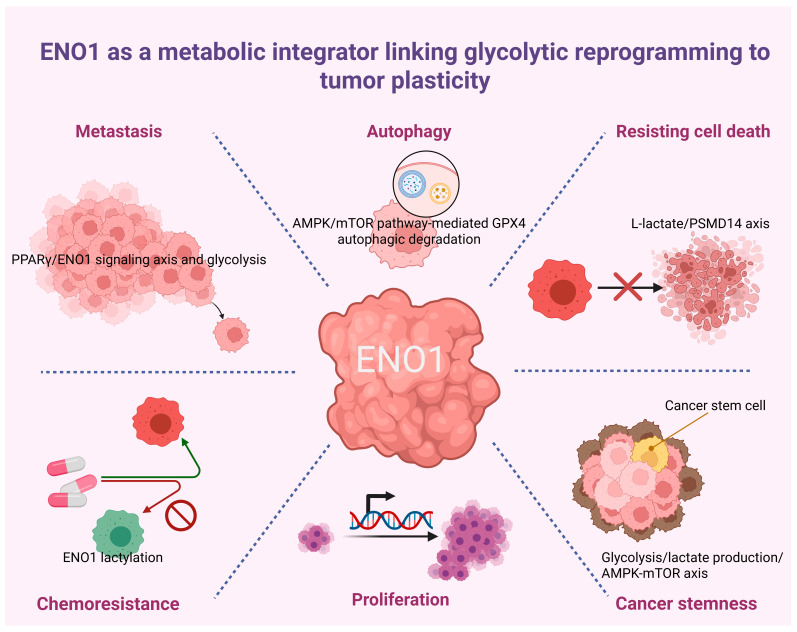
Representative scheme of the ENO1-related hallmarks of cancer. The scheme was created with BioRender, Tsung-Chieh, Lin (2026) https://www.biorender.com/ (accessed on 12 May 2026).

ENO1 has emerged as much more than a housekeeping glycolytic enzyme in cancer biology. As summarized in this review, accumulating evidence supports a broad role for ENO1 in tumor progression across multiple cancer types. Beyond its canonical function in glycolysis, ENO1 appears to participate in a wide spectrum of malignant processes, including proliferation, apoptosis resistance, stemness maintenance, autophagy-associated adaptation, metastasis, drug resistance, and angiogenesis-related microenvironmental remodeling (Table 3 and Figure 5). In parallel, pan-cancer analyses suggest that ENO1 expression is frequently dysregulated in human malignancies and may carry prognostic value in selected tumor contexts, further supporting its relevance in cancer pathobiology. A major concept emerging from the current literature is that ENO1 acts as an integrative node linking metabolic reprogramming to tumor-promoting cellular behavior. In the reviewed studies, ENO1 was repeatedly associated with enhanced glycolytic flux, increased survival under stress, and proliferative signaling support. These metabolic functions appear to extend into more aggressive phenotypes, as ENO1 was also linked to cancer stem cell-like traits, epithelial–mesenchymal transition-associated programs, invasive capacity, and metastatic dissemination. Importantly, the article also highlights that ENO1-related functions are not limited to intracellular metabolism. Extracellular and vesicle-associated ENO1, as well as autophagy-dependent ENO1 release, point to a broader role in shaping tumor–microenvironment interactions, immune modulation, and potentially angiogenic niches. Another important message is that the biological significance of ENO1 is highly context dependent. Although elevated ENO1 is commonly associated with aggressive disease behavior, the direction and magnitude of its clinical impact may vary by tumor type, disease stage, therapeutic setting, and possibly subcellular localization. Likewise, the evidence summarized here suggests that ENO1 influences treatment responses through several distinct mechanisms, including metabolic buffering, redox adaptation, EMT-related plasticity, and nucleotide metabolism. At the same time, tumors with ENO1 deletion may create unique collateral lethality-based therapeutic opportunities, indicating that both ENO1 overexpression and ENO1 loss can be clinically informative under different settings. Despite these advances, several questions remain unresolved. First, the precise upstream regulators and downstream effectors of ENO1 require clearer mechanistic definitions in individual cancer types. Second, the relative contribution of cytosolic, membrane-associated, and extracellular ENO1 should be systematically distinguished because these compartments may mediate different biological functions. Third, the prognostic and predictive value of ENO1 needs validation in larger, well-annotated clinical cohorts with standardized detection methods. Finally, the translational potential of ENO1-targeted strategies deserves further investigation, particularly in biomarker-guided settings and in combination with chemotherapy, radiotherapy, or immunotherapy. Overall, the available evidence supports ENO1 as a multifunctional and context-dependent regulator of cancer progression. While ENO1 may represent a biomarker or therapeutic candidate in selected tumor types, its clinical utility requires further validation through standardized detection methods, mechanistic studies, and prospective or well-annotated clinical cohorts.

The next stage of ENO1 research should move from association to validation. Mechanistic studies should distinguish intracellular from surface or extracellular ENO1, define the functional consequences of ENO1 post-translational modifications, and use rescue experiments to confirm causality. Clinically, ENO1 should be assessed using standardized assays, spatial localization, matched tumor–normal comparisons, and multivariable models incorporating tumor stage, treatment exposure, immune infiltration, and metabolic phenotype. Translational studies should determine whether ENO1-targeted approaches are most effective as monotherapies, metabolic sensitizers, immune modulators, or combination partners with chemotherapy, radiotherapy, targeted therapy, or immunotherapy. In this framework, the value of ENO1 is not that it represents a universal cancer biomarker, but that it may identify specific metabolic and microenvironmental vulnerabilities in selected tumor contexts.

## Figures and Tables

**Table 1:** *ENO1* mutations in a pan-cancer study of whole genomes.

Cancer Type	Protein Change	Mutation Type	Variant Type	Copy Number	Mutations in Sample
Breast Cancer	N161I	Missense_Mutation	SNP	Diploid	32
Renal Cell Carcinoma	I381F	Missense_Mutation	SNP	ShallowDel	11
Colorectal Cancer	G16R	Missense_Mutation	SNP	ShallowDel	95

SNP, single-nucleotide polymorphism.

**Table 2:** Correlation between *ENO1* and cancer patient prognosis.

Cancer Type	Prognosis	Endpoint	*p* Value	Case	Dataset	Method	Probe ID
Glioma	Poor	Overall survival	<0.001	153	TCGA	RNA Seq	
Thyroid Cancer	-	Overall survival	N.S.	501	TCGA	RNA Seq	
Lung Cancer	Poor	Overall survival	0.024	994	TCGA	RNA Seq	
Colorectal Cancer	Good	Overall survival	0.036	597	TCGA	RNA Seq	
Head and Neck Cancer	Poor	Overall survival	0.018	499	TCGA	RNA Seq	
Stomach Cancer	-	Overall survival	N.S.	354	TCGA	RNA Seq	
Liver Cancer	Poor	Overall survival	<0.001	365	TCGA	RNA Seq	
Pancreatic Cancer	Poor	Overall survival	0.017	176	TCGA	RNA Seq	
Renal Cancer	-	Overall survival	N.S.	877	TCGA	RNA Seq	
Urothelial Cancer	Poor	Overall survival	0.0014	406	TCGA	RNA Seq	
Prostate Cancer	-	Overall survival	N.S.	494	TCGA	RNA Seq	
Testis Cancer	-	Overall survival	N.S.	134	TCGA	RNA Seq	
Breast Cancer	Poor	Overall survival	0.0065	1075	TCGA	RNA Seq	
Cervical Cancer	Poor	Overall survival	0.0095	291	TCGA	RNA Seq	
Endometrial Cancer	-	Overall survival	N.S.	541	TCGA	RNA Seq	
Ovarian Cancer	-	Overall survival	N.S.	373	TCGA	RNA Seq	
Melanoma	-	Overall survival	N.S.	102	TCGA	RNA Seq	
Breast Cancer	Poor	Relapse-free survival	<0.001	4929	Datasets annotated in Kaplan–Meier plotter database (Supplementary data (Table S1))	Array	201231_s_at
Ovarian Cancer	-	Progression-free survival	N.S.	1435	Datasets annotated in Kaplan–Meier plotter database (Supplementary data (Table S1))	Array RNA Seq	201231_s_at
Lung Cancer	-	Post-progression survival	N.S.	344	Datasets annotated in Kaplan–Meier plotter database (Supplementary data (Table S1))	Array RNA Seq	201231_s_at
Gastric Cancer	Poor	Post-progression survival	0.0026	498	Datasets annotated in Kaplan–Meier plotter database (Supplementary data (Table S1))	Array	201231_s_at

Survival data were collected from the Human Protein Atlas database, the TCGA dataset, and the Kaplan–Meier plotter database. N.S., no significance; Poor, poor prognosis; Good, good prognosis.

**Table 3:** Summary of ENO1 expression, prognosis, and tumor-associated functions in cancer.

Cancer Type	ENO1 Expression	Prognosis	Tumor Functions
Lung Cancer	LUAD high in Figure 4; ENO1 IHC in LUAD [1]	Lung cancer: poor OS; PPS N.S. (Table 2)	Proliferation and invasion [1,42]; stemness [47]; osimertinib resistance in LUAD [65]; M2 polarization [60]
Intrahepatic Cholangiocarcinoma	High expression/stabilization [17]	Intrahepatic cholangiocarcinoma: poor prognosis [17]	Ferroptosis resistance [17]; AKT activation and immune evasion [62]
Breast Cancer	TNBC ENO1 stabilization [18,19]; altered sublocalization [27]	Breast cancer: poor OS and RFS (Table 2)	Glycolysis [18,19,44]; metastasis [19,54,55]; ferroptosis [45]; radiosensitivity [19]; hypoxia-related angiogenesis [44,68]
Liver Cancer	HCC HBx-induced expression [4]; altered ubiquitination/degradation [20]	Liver cancer: poor OS (Table 2)	Proliferation and apoptosis resistance [4,36,37]; glycolysis [20]; metastasis [4,53]; cholesterol metabolism [6]; immunotherapy response [20]
Colorectal Cancer	ENO1 stabilization [5]	Colorectal cancer: good OS (Table 2)	Glycolysis [5,59]; EMT and 5-FU resistance [49]; metastasis [56,58]; immune evasion [57]
Gastric Cancer	ENO1-driven glycolysis [38,39]; SENP1-mediated stabilization [22]	Stomach cancer: OS N.S.; gastric cancer: poor PPS (Table 2)	Proliferation [39,40]; apoptosis resistance [40]; chemoresistance [22,38,64]; stemness [50]
Urothelial Cancer	BLCA high in Figure 4; ENO1 stability in bladder cancer [21]	Urothelial cancer: poor OS (Table 2)	Glycolysis, migration, and invasion [21]; PD-L1 regulation [29]
Cervical Cancer	SIX1-mediated ENO1 upregulation [23]	Cervical cancer: poor OS (Table 2)	Glycolysis and proliferation [23]
Pancreatic Cancer	High ENO1 expression [28]; ENO1/PGM2L1 co-expression [63]	Pancreatic cancer: poor OS (Table 2)	Gemcitabine resistance [28,66]; migration, invasion, and glycolysis [63]
Multiple Myeloma	ENO1 linked to tumorigenicity [43]		Cell cycle, apoptosis resistance, mitophagy, and bortezomib resistance [43]
Acute Myeloid Leukemia	AML high in Figure 4; high ENO1 in early malignant differentiation [48]		Stem-cell self-renewal, chemoresistance, and differentiation blockade [48]
Glioma	GBM high in Figure 4; LGG low in Figure 4	Glioma: poor OS (Table 2)	Autophagy, chemoresistance, TME remodeling in GBM [51]; collateral lethality [10]
Prostate Cancer	PRAD low in Figure 4	Prostate cancer: OS N.S. (Table 2)	Angiogenesis-related TME effects [69]; enzalutamide sensitivity in bone metastasis [67]
Osteosarcoma	High ENO1 at lung metastatic sites [61]		Metabolic reprogramming, migration, invasion, and metastasis [61]
Ewing Sarcoma	Circulating ENO1-positive EVs [24]		Circulating biomarker potential [24]
Diffuse Large B-cell Lymphoma	ENO1-related gene signature [26]	Diffuse large B-cell lymphoma: worse outcome [26]	Prognosis and therapeutic-response prediction [26]
Peripheral T-cell Lymphoma			Ferroptosis and metabolic remodeling [46]
Chronic Myeloid Leukemia			TKI sensitivity, ferroptosis, and GPX4 autophagic degradation [52]
Ovarian Cancer	OV high in Figure 4	Ovarian cancer: OS N.S.; PFS N.S. (Table 2)	
Melanoma	SKCM high in Figure 4	Melanoma: OS N.S. (Table 2)	
Testis Cancer	TGCT high in Figure 4	Testis cancer: OS N.S. (Table 2)	
Thyroid Cancer	THCA low in Figure 4	Thyroid cancer: OS N.S. (Table 2)	
Renal Cancer	KICH low in Figure 4	Renal cancer: OS N.S. (Table 2)	
Head and Neck Cancer		Head and neck cancer: poor OS (Table 2)	
Endometrial Cancer		Endometrial cancer: OS N.S. (Table 2)	
Uveal Melanoma	UVM high in Figure 4		
Mesothelioma	MESO high in Figure 4		
Uterine Carcinosarcoma	UCS high in Figure 4		
Pheochromocytoma and Paraganglioma	PCPG low in Figure 4		

Note: Cancer type names in the first column were standardized using broad disease categories when survival data were derived from pan-cancer or clinical outcome analyses. Specific histological subtypes or TCGA entities are indicated in the expression column when applicable. Figure 4 status was summarized according to the manuscript description. Prognostic information derived from the manuscript survival table is indicated as “Table 2” in the prognosis column. Empty cells indicate that the corresponding information was not specifically described in the manuscript text or tables. Abbreviations: 5-FU, 5-fluorouracil; AML, acute myeloid leukemia; BLCA, bladder urothelial carcinoma; EVs, extracellular vesicles; GBM, glioblastoma multiforme; HCC, hepatocellular carcinoma; IHC, immunohistochemistry; KICH, kidney chromophobe; LGG, lower-grade glioma; LUAD, lung adenocarcinoma; MESO, mesothelioma; OS, overall survival; OV, ovarian serous cystadenocarcinoma; PCPG, pheochromocytoma and paraganglioma; PFS, progression-free survival; PPS, post-progression survival; PRAD, prostate adenocarcinoma; RFS, relapse-free survival; SKCM, skin cutaneous melanoma; TGCT, testicular germ cell tumor; THCA, thyroid carcinoma; TNBC, triple-negative breast cancer; UCS, uterine carcinosarcoma; UVM, uveal melanoma. Abbreviations already defined before Table 3 are not repeated.

## Data Availability

No new data were created or analyzed in this study. Data sharing is not applicable to this article.

## Supplementary Materials

The following supporting information can be downloaded at: https://www.mdpi.com/article/10.3390/ijms27104479/s1.
