# Supplementary material for: ENO1 as a Central Regulator Linking Metabolic Reprogramming to Tumor Plasticity
Source: Int J Mol Sci. 2026 May 16;27(10):4479. doi: 10.3390/ijms27104479 (PMC13208067; doi:10.3390/ijms27104479)
Supplement: Supplementary file 1 [file ijms-27-04479-s001.zip › ijms-4297409-supplementary.pdf]

Supplementary Table S1

Datasets included in specific cancer type in Kaplan-Meier plotter database

Breast cancer	E-MTAB-365, E-TABM-43, GSE: 11121, 12093, 12276, 1456, 16391, 16446, 16716, 17705, 17907, 18728, 19615, 20194, 20271, 2034, 20685, 20711, 21653, 22093, 25066, 2603, 26971, 29044, 2990, 31448, 31519, 32646, 3494, 36771, 37946, 41998, 42568, 43358, 43365, 45255, 4611, 46184, 48390, 50948, 5327, 58812, 61304, 65194, 6532, 69031, 7390, 76275, 78958, 9195
Ovarian cancer	GSE: 14764, 15622, 18520, 19829, 23554, 26193, 26712, 27651, 30161, 3149, 51373, 63885, 65986, 9891, TCGA (N=565)
Lung cancer	CAARRAY, GSE: 14814, 19188, 29013, 30219, 31210, 3141, 31908, 37745, 43580, 4573, 50081, 8894, TCGA (N=133)
Gastric cancer	GSE: 14210, 15459, 22377, 29272, 51105, 62254
